# Epigenetic Activation and Silencing of the Gene that Encodes IFN-γ

**DOI:** 10.3389/fimmu.2013.00112

**Published:** 2013-05-16

**Authors:** Thomas M. Aune, Patrick L. Collins, Sarah P. Collier, Melodie A. Henderson, Shaojing Chang

**Affiliations:** ^1^Department of Medicine, Vanderbilt University School of MedicineNashville, TN, USA; ^2^Department of Pathology, Microbiology and Immunology, Vanderbilt University School of MedicineNashville, TN, USA

**Keywords:** interferon-gamma, T helper cells, natural killer cells, natural killer T cells, epigenetics, CpG methylation, long non-coding RNA

## Abstract

Transcriptional activation and repression of genes that are developmentally regulated or exhibit cell-type specific expression patterns is largely achieved by modifying the chromatin template at a gene locus. Complex formation of stable epigenetic histone marks, loss or gain of DNA methylation, alterations in chromosome conformation, and specific utilization of both proximal and distal transcriptional enhancers and repressors all contribute to this process. In addition, long non-coding RNAs are a new species of regulatory RNAs that either positively or negatively regulate transcription of target gene loci. IFN-γ is a pro-inflammatory cytokine with critical functions in both innate and adaptive arms of the immune system. This review focuses on our current understanding of how the chromatin template is modified at the *IFNG* locus during developmental processes leading to its transcriptional activation and silencing.

## T Helper Cell Differentiation

The cytokine, IFN-γ, plays key roles in controlling infection by intracellular pathogens, including bacteria and viruses, as well as malignant transformation and growth (Mosmann and Coffman, 1989; Dunn et al., 2004). Two major sources of IFN-γ include T cells and NK/NKT cells (Biron et al., 1999). Once NK/NKT cells immigrate to the periphery, they are fully competent to produce IFN-γ in response to a variety of extracellular stimuli. In contrast, once T cells immigrate to the periphery, they must endure additional developmental programs before they are fully competent to produce large quantities of IFN-γ. For the most part, developmental programs in T cells are driven by a combination of T-cell receptor signaling via antigenic stimulation and by cytokines, in particular IL-12, produced by the innate immune system (Zhu et al., 2010). Key transcription factors that drive the differentiation process include Stat4 and T-bet (Thierfelder et al., 1996; Szabo et al., 2000; Mullen et al., 2002). Differentiated T cells, termed T helper 1 (Th1) and T cytotoxic 1 (Tc1) produce IFN-γ as their predominant cytokine in response to secondary antigenic stimulation.

At the chromatin level, this differentiation process arises from marked epigenetic changes spanning a region greater than 100 kb surrounding the gene that encodes IFN-γ (Zhou et al., 2004; Chang and Aune, 2005; Schoenborn et al., 2007). These epigenetic changes include gain and loss of histone modifications associated with activation and silencing of gene transcription, respectively, changes in DNase hypersensitivity sites, and changes in methylation (Me) of CpG dinucleotides, a modification associated with transcriptional silencing. In simple terms, a naïve T cell can be considered a pluripotent cell capable of differentiating into multiple lineages, including Th1, Th2 or Th17, and other variants (Weaver et al., 2007). Upon completion of this differentiation pathway achieved through epigenetic changes to the chromatin, differentiated cells become fully capable of transcribing their signature cytokine, e.g., *IFNG*, and silencing the cytokine genes transcribed by the other lineages, *IL4*, *IL13* (Th2), or *IL17* (Th17) (for simplicity, the human gene symbol convention will be used throughout, all letters capitalized and italicized, known differences between mouse and human will be highlighted in the text).

## Histone Code

Developmental processes represent heritable phenotypic changes passed on to daughter generations in the absence of changes in the genetic code. Th1/Th2 differentiation is an example of this process. Thus, the term epigenetics arose to define these heritable changes in gene expression leading to changes in phenotype in the absence of changes in the underlying genetic code. The histones that wrap the DNA into the chromatin fiber play key roles in these epigenetic changes. The amino terminal “tails” of the core histones, H2A, H2B, H3, and H4 undergo enzymatic post-translational modifications including Me, acetylation (Ac), phosphorylation, ubiquitination, and sumoylation and these modifications are associated with activation and silencing of specific genes (Rea et al., 2000; Strahl and Allis, 2000; Turner, 2002). This recognition gave rise to the histone code hypothesis proposing that these histone modifications at gene loci are critical for cell-type and stimulus-specific transcriptional activation and silencing of genes. The general view is that transcription factors that bind to specific gene loci recruit the enzymes that catalyze formation of these histone “marks” to the loci thus establishing specific cell-type and stimulus-specific patterns of histone “marks.” Initially it was thought that these modifications were permanent once formed. However, the discovery of enzymes that also remove these “marks” has changed this view and suggests that epigenetics may represent a very dynamic process.

The *IFNG* locus undergoes complex patterns of histone modifications in response to Th1/Th2 differentiation signals (Zhou et al., 2004; Chang and Aune, 2005, 2007; Schoenborn et al., 2007). The *IFNG* locus is relatively devoid of histone “marks,” in particular H4-Ac “marks,” in proliferating CD4+ T cells in the absence of Th1/Th2/Th17 polarizing signals, Th0 cells. However, addition of a specific inhibitor of histone deacetylases produces an H4-Ac pattern across the *IFNG* locus and active *IFNG* transcription similar to that observed in effector Th1 cells. This has been interpreted to mean that both histone acetyltransferases and histone deacetylases are recruited to the *IFNG* locus in Th0 cells resulting in no accumulation of H4-Ac “marks.” Inhibition of histone deacetylases allows histone acetyltransferase-catalyzed accumulation of H4-Ac and *IFNG* transcription (Chang et al., 2008; Aune et al., 2009).

Stat4, Runx3, T-bet, and multiple other transcription factors are recruited to the promoter and multiple other conserved non-coding sequence (CNS) elements across the *IFNG* locus in response to Th1 polarizing signals (Zhu et al., 2010). Th1 differentiation also stimulates formation of a complex pattern of H4-Ac and H3K4-Me “marks” across the locus, modifications that promote transcription. In part, accumulation of H4-Ac across the locus is dependent upon T-bet mediated expulsion of histone deacetylases from the locus. Presumably these transcription factors also recruit additional histone acetyltransferases and histone methyltransferases to the locus to form this complex epigenetic pattern. Formation of this complex pattern of epigenetic modifications is largely abrogated in the absence of Stat4 or T-bet. In large part, these epigenetic modifications are sustained in both memory CD4 and CD8 T cells (Northrop et al., 2006; Schoenborn et al., 2007). Interestingly, differentiation of memory CD8 T cells requires CD4 T cell “help” and this “helper” function is revealed by the requirement for CD4 T cell help at the level of epigenetic modifications at the *IFNG* locus in memory CD8 T cells.

In contrast to CD4 T cells, NK cells do not need to endure additional developmental programs once they immigrate to the periphery to rapidly respond to extracellular stimuli and transcribe *IFNG*. Peripheral NK cells also possess a pre-existing H4-Ac pattern of epigenetic modifications across the *IFNG* locus and presumably H3K4-Me modifications (Chang and Aune, 2005). This pattern is similar but not identical to that seen in either effector Th1 or Tc1 cells differentiated in tissue culture in response to polarizing signals or *in vivo*. In NK cells, the *IFNG* locus is further modified by H4-Ac at additional sites across the locus in response to stimuli that induce *IFNG* transcription. Thus, the differentiation signals driving NK cell development also produce epigenetic modifications across the *IFNG* locus that presumably allow NK cells to respond to extracellular stimuli by rapidly transcribing *IFNG*.

Th2 differentiation signals also initiates epigenetic modifications across the *IFNG* locus (Chang and Aune, 2007). In contrast to the “marks” formed in response to Th1 differentiation signals, the marks formed in response to Th2 differentiation are repressive H3K27 di- and tri-Me “marks.” Consequently, silencing of *IFNG* in Th2 cells, like transcriptional activation of *IFNG* in Th1 cells, is an active process. These “marks” are formed at approximately the same CNS’s that are modified in Th1 by activating “marks.” Further, key transcription factors that drive the Th2 differentiation process, Stat6 and GATA-3 (Kaplan et al., 1996; Zheng and Flavell, 1997), are recruited to the *IFNG* locus and are required to recruit EZH2, the enzyme that catalyzes H3K27-Me, to the locus. Thus, Stat6 and GATA-3 are recruited to both the *IFNG* and *IL4* loci in developing Th2 cells. However, somehow these transcription factors recruit enzymes that catalyze formation of activating histone “marks” to the *IL4* locus and enzymes that catalyze formation of repressive histone “marks” to the *IFNG* locus. Thus, Stat6 and GATA-3 are both activating and repressive transcription factors depending upon the gene locus in question.

Another form of histone modification is H3K9 Me. HeK9 Me is generally associated with transcriptional repression. However, there is also evidence that H3K9 Me occurs at gene loci that are actively transcribed (Vakoc et al., 2005). The *IFNG* locus is also modified by H3K9 Me in response to T-cell activation and T helper cell differentiation in developing Th0, Th1, and Th2 cells (Chang and Aune, 2007). In Th0 cells and developing Th1 cells, these “marks” are sustained. In contrast, in developing Th2 cells, these marks are extinguished and replaced by H3K27-Me marks. A general view is that the H3K27-Me “mark” is associated with cell-type specific and stimulus-specific active transcriptional repression while the H3K9 Me “mark” is associated with silencing of genes in cells that lack the potential to transcribe a given gene. One possible interpretation is that H3K9 Me serves to repress *IFNG* transcription in Th0 cells but also to dampen *IFNG* transcription in effector Th1 cells. In principle, this adds another layer of control to *IFNG* transcription. This could be important since IFN-γ is such a potent cytokine and excess IFN-γ production may have deleterious consequences such as excess inflammation and autoimmunity. These results also represent a clear example of the dynamic nature of histone modifications in differentiating cells that further our understanding of the execution of the histone code.

## DNA Methylation

DNA Me of CpG dinucleotides is a second major epigenetic mechanism to achieve silencing of transcription of specific genes during developmental processes (Okano et al., 1999; Cedar and Bergman, 2009). In naïve CD4 T cells, CpG dinucleotides within the *IFNG* introns and most, but not all *IFNG* distal CNS regions, and not the promoter, are heavily methylated (Schoenborn et al., 2007). Me is largely but not completely lost during Th1 differentiation. In contrast, DNA Me is largely sustained in response to Th2 and Th17 differentiation signals. Further, the *IFNG* promoter also becomes hyper-methylated in these opposing lineages. Moreover, lineage specificity of effector T helper cells exhibits plasticity during the differentiation process such that early during the differentiation process effector Th1 cells can be converted to effector Th2 or Th17 if exposed to those stimuli that drive Th2 or Th17 differentiation. The converse is also true for effector Th2 and Th17 cells. Ultimately, these effector cells become permanently locked into their respective Th1, Th2, or Th17 phenotypes.

The pattern of DNA Me at the human *IFNG* locus is somewhat different than the mouse (Janson et al., 2008; Dong et al., 2013). Thus, both the *IFNG* promoter and a proximal conserved nucleotide sequence, termed CNS-1, are methylated in naïve T helper cells and Me is lost as T helper cells progress through their differentiation paths to effector cells and memory cells. Evidence suggests that the Me/de-Me status of *IFNG* may be clinically relevant. *IFNG* is inappropriately hyper-methylated in tumor-infiltrating lymphocytes in colon cancer, which may represent a form of tumor-induced immunosuppression.

Comparing murine naïve CD4 T cells to NK cells demonstrates a further distinction in DNA Me patterns at *IFNG*. Thus, the *IFNG* first intron is heavily methylated in naïve CD4 T cells while it is largely un-methylated in effector Th1 cells and NK cells (Tato et al., 2004). Thus, as with histone Ac patterns at the *IFNG* locus, lack of DNA Me at the *IFNG* locus in NK cells is consistent with their ability to respond to external stimuli by rapidly producing IFN-γ.

**Figure 1:**
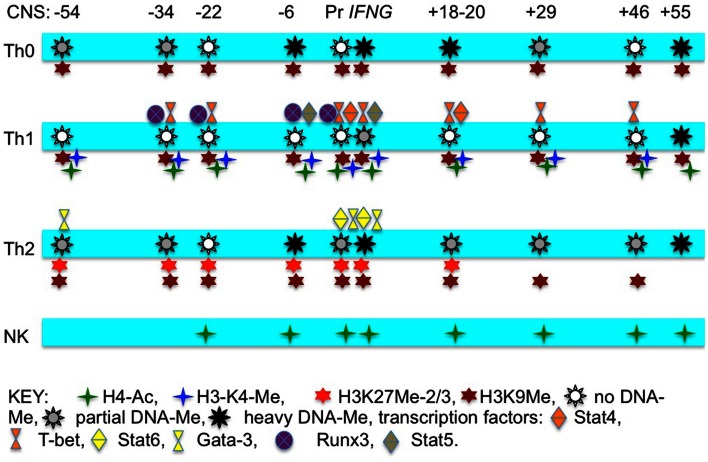
**Schematic illustrating transcription factors that bind to individual CNS across the *IFNG* locus, histone “marks” across the locus, and degree of CpG dinucleotide methylation at the different CNS in T cell and NK cell lineages**.

There are several DNA methyltransferases (DNMT) responsible for DNA Me. It is generally thought that DNMT1 is necessary to maintain DNA Me at the *IFNG* locus in CD4 T cells in the undifferentiated state (Wilson et al., 2002). In contrast, DNMT3a catalyzes DNA Me of the *IFNG* promoter in response to Th2 and Th17 differentiation signals to sustain *IFNG* silencing in these opposing lineages even if re-exposed to Th1 differentiation signals (Thomas et al., 2012). Thus, different DNMT’s are utilized to silence *IFNG* transcription in naïve CD4 T cells and probably in non-lymphoid cells than are utilized to silence *IFNG* transcription in effector CD4 T cells of opposing lineages. A summary of known transcription factors that bind to the *IFNG* locus and the different epigenetic marks in T cells and NK cells is shown in Figure 1.

## Enhancers

Analysis of epigenetic modifications, DNase hypersensitivity sites, histone modifications, and CpG Me, has produced a “roadmap” of the *IFNG* locus and has shown how this “roadmap” changes in cells as they differentiate to actively transcribe or actively silence *IFNG*. However, it cannot be inferred from these analyses whether CNS’s undergoing epigenetic modifications in response to these differentiation signals contribute to transcriptional activation or repression and how this is orchestrated. Various strategies have been employed to dissect functional properties of CNS’s across the *IFNG* locus undergoing epigenetic modifications in response to differentiation signals. These include analysis of enhancer activity by using reporter constructs in cell lines, use of these reporter constructs as transgenes in mice, and use of bacterial artificial chromosomes (BAC) as transgenes in mice (Soutto et al., 2002; Shnyreva et al., 2004; Tong et al., 2005; Hatton et al., 2006; Schoenborn et al., 2007; Collins et al., 2010, 2012b). Typically, these BAC transgenes have *IFNG* positioned in the middle of the BAC and contain 50–100 kb of 5′ and 3′ sequence. Thus, they contain the CNS’s that undergo epigenetic modifications in response to the developmental signals that drive lineage choice.

Expression of IFN-γ by T cells can be thought of as proceeding through discrete stages of development and this is recapitulated in tissue culture models. Naïve T cells rapidly produce very low levels of IFN-γ and IL-4 in response to TCR stimulation. The combination of TCR stimulation and IL-12 will initiate the differentiation process resulting in production of large quantities of IFN-γ after several days in culture and this is also observed *in vivo*. Effector Th1 cells are defined by their capacity to rapidly produce large quantities of IFN-γ after a secondary TCR stimulation in the absence of other stimuli, such as IL-12. This differentiation process requires 4–7 days in most tissue culture models, which is similar to that observed, *in vivo*. The distinction between effector Th1 cells and memory effector Th1 cells is that memory effector Th1 cells are long-lived cells that exist in the periphery and rapidly respond to secondary antigenic stimulation to produce large quantities of IFN-γ. As described above, NK and NKT cells in the periphery respond to a variety of extracellular stimuli by rapidly producing large quantities of IFN-γ.

Thus, these different stages of differentiation that T cells endure make it possible to ascertain if different CNS’s possess common functions or unique functions. For example, the function of CNS-30, positioned 30 kb upstream of human *IFNG*, has only been studied using the BAC transgenic system. Deletion of CNS-30 abrogates *IFNG* expression in developing Th1 cells, effector Th1 cells, and memory Th1 cells. This CNS binds the transcription factor, Runx3, and is required for RNA Pol2 recruitment to the promoter providing an underlying mechanism for the requirement of this CNS for active *IFNG* transcription. CNS-30 is also required for *IFNG* expression by NKT cells but not by NK cells. One unexpected finding is that deletion of CNS-30 does not impact writing of the histone code in T cells. Thus, formation of histone marks across the locus is not impacted by deletion of CNS-30 while *IFNG* transcription is severely compromised.

The general view is that the conserved DNA sequence of a CNS implies function. A corollary of this argument is that CNS’s in different species should have the same function. The mouse CNS-22 and the human CNS-16 are evolutionary homologs. However, their function is quite different. Mouse CNS-22 is necessary for *IFNG* transcription during development of murine Th1 effector cells. In contrast, human CNS-22 is not necessary for *IFNG* transcription during development of human Th1 cells. In fact, human CNS-22 possesses repressor function and deletion of human CNS-22 results in active *IFNG* transcription in developing and effector Th2 cells under conditions where *IFNG* transcription should be actively repressed. These results are consistent with the idea that conservation of DNA sequence implies function but demonstrate that evolutionary CNS homologs do not necessarily possess identical function.

Conserved non-coding sequence-2 (CNS-6 in mice) plays a key role in early epigenetic remodeling of the *IFNG* locus in response to Th1 differentiation signals (Shi et al., 2008). Initially, a Jak3-dependent cytokine signal, probably IL-2 stimulates recruitment of Stat5 to this CNS. NFAT is also recruited to this CNS. These early signals are thought to facilitate T-bet recruitment to the promoter and subsequent epigenetic modifications. Reporter assays reveal that CNS-2 possesses enhancer function. However, in contrast to its role in directing early epigenetic changes at the *IFNG* locus, CNS-2 is not required for active *IFNG* transcription by developing effector Th1 cells. CNS-2 is necessary for active transcription by both effector Th1 cells and effector memory Th1 cells. Thus, CNS-2 orchestrates early epigenetic events during Th1 differentiation and is absolutely required for *IFNG* transcription in response to TCR stimulation by differentiated Th1 cells of both effector and memory lineages.

An additional CNS positioned 3′ of *IFNG*, CNS + 18–20, has been examined in both reporter assays and in BAC transgenic models. In reporter assays, this CNS lacks enhancer activity but cooperates with CNS-2 to increase CNS-2 enhancer activity (Shnyreva et al., 2004). Analysis of a deletion of CNS + 18–20 in the BAC transgenic system reveals that this CNS is required for production of IFN-γ by effector memory Th1 cells but not by developing Th1 or effector Th1 cells that have differentiated, *in vitro* (Collins et al., 2012a). Taken together, these results also demonstrate that distinct CNSs are required to transcribe *IFNG* at each stage of the Th1 differentiation pathway.

**Figure 2:**
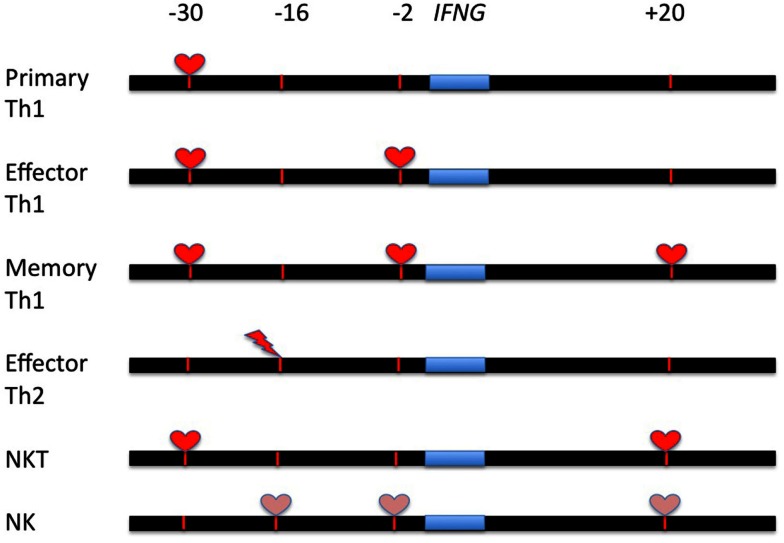
**Summary of the regulation of *IFNG* transcription by distal CNS at different stages of development in T cells and NK/NKT cells**. Red hearts identify CNS’s that are absolutely required for *IFNG* transcription in the indicated cell lineage; pink hearts identify CNS’s that are partially required for *IFNG* transcription in the indicated cell lineage. Red lightning strikes identify CNS’s that repress *IFNG* transcription.

Analysis of CNS requirements for *IFNG* transcription by NK and NKT cells further supports the notion that CNS functions are not identical in all cells. NKT cells require both CNS-30 and CNS + 18–20 to produce IFN-γ independent of whether or not they are stimulated via the TCR (a-galactosyl ceramide) or by the cytokine combination, IL-12 and IL-18. NKT cells do not require CNS-2 for active *IFNG* transcription. In contrast, NK cells do not exhibit an absolute requirement for any individual CNS but rather exhibit partial requirements for CNS-16, CNS-2, and CNS + 18–20 to produce IFN-γ. Thus, CNS-16 is a bi-functional enhancer repressing *IFNG* transcription in Th2 cells yet activating *IFNG* transcription in NK cells. Another feature of this modular design is that defects in transcription revealed by CNS deletion are independent of stimulus, TCR stimulation versus cytokine stimulation (IL-12 and IL-18) but are cell-type specific. A schematic of different CNS functions in T cells and NK cells is shown in Figure 2.

## Shared Enhancers

Although *IL26* is deleted from rodent genomes, linkage of *IL26* and *IFNG* is preserved throughout evolutionary time from zebrafish to humans (Dumoutier et al., 2000). Physical linkage of genes over evolutionary time is generally thought to confer fitness to a species yet why gene order may confer fitness to a species is incompletely understood (Lercher et al., 2002, 2003). For example, Th17 lineages express *IL26* and Th1 lineages express *IFNG* so *IL26* and *IFNG* should not be considered as part of a related gene family. An alternate hypothesis would be that distal enhancer elements may be shared between adjacent genes, which would require linkage be maintained throughout evolution and this was possible to test experimentally using the BAC transgenic model with deletions of specific CNSs. One enhancer distal to the *IL26* promoter and −77 kb from *IFNG* is uniquely required for *IL26* expression but not for *IFNG* expression (Collins et al., 2012b). In contrast, the CNS-30 enhancer element positioned between *IL26* and *IFNG* is required for both *IL26* and *IFNG* expression. One function of this enhancer is to facilitate recruitment of RNA pol II to promoters of both genes. Thus, sharing of distal enhancers between adjacent genes may contribute to evolutionary preservation of gene order.

Taken together, these studies illustrate how different cells employ distal CNS’s to regulate transcription. Perhaps NK cells represent the simplest pattern where multiple CNS’s cooperate to drive *IFNG* transcription. Deletion of a single CNS is not sufficient to abrogate transcription. In contrast, NKT cells utilize two distal CNS’s and both are absolutely essential for *IFNG* transcription. CD4 T cells exhibit a somewhat more complex usage of distal CNS where one, two or three CNS’s are necessary to drive *IFNG* transcription depending upon their stage of differentiation; developing Th1 cells, effector Th1 cells, or memory Th1 cells. As far as it has been examined, individual CNS’s do not seem to be required to write or sustain the histone code. Rather they seem necessary to recruit RNA Pol II to the promoter to initiate transcription of *IFNG*.

## *IFNG* Transcription and the Long Non-Coding RNA, *TMEVPG1* (*NeST*)

Approximately 1% of the genome is devoted to transcription of protein-coding genes. Surprisingly, results from the ENCODE project have identified multiple new species of RNA and demonstrate that the majority of the genome is transcribed in some cell at some point in development. One new species of RNA has been termed long non-coding RNA (lncRNA) (Guttman et al., 2009). LncRNAs may be either intragenic or intergenic. They may consist of a single exon or multiple exons and introns that are spliced into a mature RNA. All are transcribed in low abundance into long segments of RNA, which vary between 200 to 2000 base pairs in length, but lack coding potential because they are littered with stop codons. In general, lncRNA genes demonstrate species to species conservation of DNA sequence. Current estimates suggest that 1000s of lncRNAs exist in the human genome but functional and molecular mechanisms have been elucidated in fewer than 10% of these lncRNAs. LncRNAs have been shown to play key roles in all developmental compartments including the immune system, as well as to a host of human diseases including cancer, cardiac disease, and neurological conditions (Ji et al., 2003; Ishii et al., 2006; Gupta et al., 2010; Kerin et al., 2012).

The major function of lncRNAs is to regulate expression of protein-coding genes. For example, the lncRNA *XIST*, in concert with nine other lncRNAs, is instrumental in the inactivation of the X chromosome, a core biological process to regulate gene dosage in females (Wutz, 2011). The lncRNAs, *HOTAIR*, and *AIR*, regulate the *HOXD* and *IGF2R* loci, respectively, and are critical for segmentation and genomic imprinting in early phases of development (Sleutels et al., 2002). *HOTAIR*, also classified as a transcriptional repressor, assembles as a molecular scaffold for histone modifying complexes containing PRC2, but is unique in that it modulates the *HOXD* locus *in trans* (Rinn et al., 2007; Tsai et al., 2010). Enhancement of the *IGF2/H19* locus through chromatin looping via CTCF binding sites requires both the transcription factor p68 and the *SRA* lncRNA (Yao et al., 2010; Nagano and Fraser, 2011). *BRAVEHEART*, a recently described lncRNA, has a significant role in terminal differentiation of myocardial cells also by associating with PRC2 complexes (Klattenhoff et al., 2013).

Long non-coding RNAs also function as molecular responders within cell signaling networks. For example, two lncRNAs termed long intergenic non-coding RNA-p21 or *lincRNA-p21* and *PANDA* (P21 associated ncRNA DNA damage activated) are located within the *CDKN1A* locus and are regulated by p53, a potent tumor suppressor. *LincRNA-p21* negatively regulates expression of pro-apoptotic genes conferring cell cycle regulation, whereas *PANDA* responds to DNA damage by sequestration of the NF-YA transcription factor resulting in cell survival (Huarte et al., 2010; Hung et al., 2011). The lncRNA, *NRON*, is a negative regulator of the transcription factor NFAT by forming protein:RNA complexes resulting in sequestration of NFAT in the cytosol (Willingham et al., 2005). Although the majority of classified lncRNAs are repressors of transcription, a small subset of enhancer lncRNAs has been described (Orom et al., 2010). *HOTTIP*, a third lncRNA regulator of the *HOX* gene family, associates with WDR5 of the MLL/MLL1 histone modifying complex to promote permissive Me marks on H3K4 at the *HOXA* locus in embryonic fibroblasts. LncRNAs have even been shown to regulate other lncRNAs, as in the case of *Jpx* and *XIST*. *Jpx* promotes expression of *XIST* *in trans* and without *Jpx*, the critical process of X chromosome inactivation does not occur (Tian et al., 2010).

Theiler’s Murine Encephalitis Virus Possible Gene 1 (*TMEVPG1*) also named NEttoie Theiler’s Pas Salmonella (*NeST*) is the first identified enhancer lncRNA of the immune system to regulate expression of a master cytokine such as IFN-γ (Vigneau et al., 2003; Collier et al., 2012; Gomez et al., 2013). Located 170 kb downstream from the *IFNG* gene and transcribed from the antisense strand relative to *IFNG*, the 33 kb *TMEVPG1* gene is spliced into a 1.7 kb RNA transcript in humans (0.9 kb in mice). Initially described in the context of an intracranial Theiler’s virus infection, the inability to control chronic viral infection in mice maps to a genetic deficiency within the *TMEVPG1* gene. Analyses of epigenetic regulatory marks across the *TMEVPG1* promoter indicate that it is an area of active transcription by enrichment of H3K9 and H4-Ac as well as H3K4 mono- and tri-Me. The *TMEVPG1* gene is conserved among placental mammals with increasing rates of sequence conservation between human and mouse orthologs within the first exon and intron. Further, several Th1-specific DNase I HS sites are found upstream to and at the *TMEVPG1* promoter supporting the permissivity of the locus to Th1-specific expression.

Functionally, *TMEVPG1* is an enhancer of *IFNG* transcription. The transcription factors, Stat4 and T-bet, are required for effector Th1 cells to express *TMEVPG1* and *TMEVPG1*, in cooperation with T-bet, stimulates *IFNG* transcription. These results are confirmed in a recent study of *TMEVPG1* in the context of a *Salmonella* infection in which *TMEVPG1* confers protection against oral infection by this pathogen. Although induction of a lncRNA by a viral pathogen has been demonstrated previously, this report is the initial demonstration of the critical role a lncRNA plays in bacterial infections. Thus, *TMEVPG1* (*NeST*) joins *HOTTIP* as a lncRNA that associates with WDR5 to promote H3K4-Me of histones, a mark associated with transcriptional activation, presumably at the neighboring *IFNG* locus.

## Looping of Chromatin Domains

It is now recognized that alterations in chromosome conformation is a common mechanism to bring chromatin domains within close proximity to one another or to exclude chromatin domains from each other (Sajan and Hawkins, 2012). In this way, distal activating CNS can be brought into close proximity to a promoter and distal repressive CNS can be excluded from the promoter of an actively transcribed gene. The *IFNG* locus is a prime example of this phenomenon. Alterations in conformation of the *IFNG* locus proceed through discrete stages. In unstimulated naïve T cells, the *IFNG* genomic locus associates with the Th2 cytokine genomic locus and exists in an open conformation (Spilianakis et al., 2005). TCR stimulation and subsequent activation and proliferation are sufficient to free the *IFNG* genomic locus from the Th2 cytokine genomic locus and to initiate alterations in the conformation of the *IFNG* locus (Eivazova and Aune, 2004). Further alterations in conformation are seen as T cells differentiate along the Th1 pathway and T-bet plays a key role in these additional changes in conformation (Hadjur et al., 2009; Sekimata et al., 2009). Thus, both Th1-independent and -dependent pathways exist that allow the *IFNG* locus to adopt a new conformation. The general view is that this new conformation recruits distal CNS with their associated transcription factors and epigenetic modifying machinery to *IFNG* to establish long-range epigenetic modifications that ultimately facilitate recruitment of RNA Pol2 to the promoter and active transcription.

**Figure 3:**
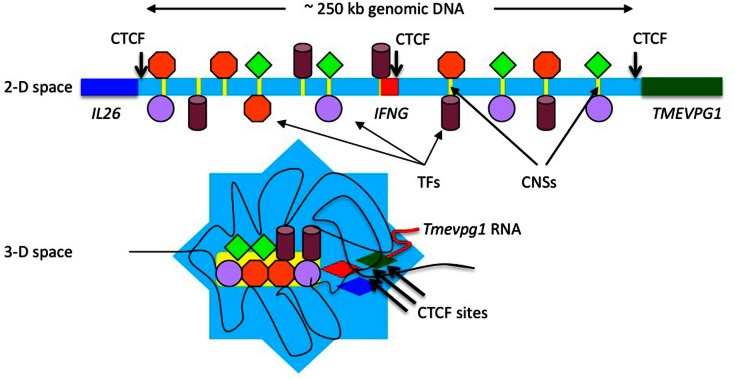
**Schematic illustration of looping of chromatin domains within the *IFNG* locus**. Changes in the three-dimensional (3-D) conformation of the *IFNG* locus may recruit distal conserved non-coding sequences (CNS) to the gene to regulate transcription. Distal evolutionarily conserved DNA elements are occupied by transcription factors after initiation of T helper type 1 (Th1)/Th2 differentiation programs. These transcription factors can tether enzymes that catalyze histone modifications, chromatin remodeling, and other functions to these DNA elements. Changes in three-dimensional conformation of the locus may serve to localize these DNA elements and their associated proteins to the *IFNG* gene. It has been argued that CTCF sites serve as boundary elements to insulate *IFNG*. An alternative hypothesis is that CTCF sites serve to bring *IL26* close to *IFNG* to allow it to share the same transcriptional enhancers and to bring *TMEVPG1* close to *IFNG* to allow *TMEVPG1* RNA to associate with the *IFNG* locus.

CCCTC-binding factor or CTCF is a zinc finger transcription factor that binds the core CCCTC sequence (Filippova et al., 1996; Rubio et al., 2008; Phillips and Corces, 2009). One role of CTCF is to regulate the three-dimensional structure of chromatin. It also functions as an insulator by defining the boundaries between euchromatin and heterochromatin. Three CTCF or CCCTC-binding factor sites are located across the *IFNG* locus and these are conserved between humans and mice (Hadjur et al., 2009; Sekimata et al., 2009). One is positioned proximal to *IL26*, one is located proximal to *TMEVPG1* and one is located within an *IFNG* intron. CTCF is typically known as a transcriptional repressor and is also involved in insulator activity and regulation of chromatin architecture (Phillips and Corces, 2009). In this way it can define boundaries between active chromatin and heterochromatin. In effector Th1 cells, the two distal CTCF sites loop into the CTCF site located within *IFNG*. It has been proposed that this defines the boundaries of the *IFNG* locus and serves to insulate *IFNG* from the negative effects of adjacent heterochromatin. However, adjacent *TMEVPG1* is co-expressed with *IFNG* in effector Th1 cells. Adjacent *IL26* is co-expressed with *IFNG* under some conditions. Thus, an alternate hypothesis is that the CTCF sites recruit adjacent *TMEVPG1* and *IL26* into the *IFNG* locus, which would serve two purposes. First, localizing the *TMEVPG1* gene close to the *IFNG* gene would permit *TMEVPG1* RNA to more easily associate with the IFNG locus. Second, localizing the *IL26* gene close to the *IFNG* gene would permit sharing of the transcriptional and enhancer machinery necessary to express both genes. Thus, these CTCF sites may not serve to insulate *IFNG* from *IL26* and *TMEVPG1* but rather to bring them into close proximity to each other to facilitate their function and co-expression. Figure 3 presents a schematic illustration of how organization of the *IL26*-*IFNG*-*TMEVPG1* locus may look in two-dimensional and three-dimensional space.

## Concluding Remarks

Over the past decade, many studies have contributed to our increased understanding of the complexities of the regulation of gene transcription. Given the fundamental roles that cytokines play in both the innate and adaptive immune systems and the fact that their activities are mostly regulated at the transcriptional level, it is easy to see how the genes that encode these proteins must be under very tight regulation. Too little production of a cytokine such as IFN-γ could fail to control pathogen infection leading to bacteremia, viremia, or even death. Conversely, excess production of cytokines such as IFN-γ could produce excess inflammation, autoimmunity, or death.

## Conflict of Interest Statement

The authors declare that the research was conducted in the absence of any commercial or financial relationships that could be construed as a potential conflict of interest.

## References

[B1] Aune T. M. Collins P. L. Chang S. (2009). Epigenetics and T helper 1 differentiation. Immunology 126, 299–305 10.1111/j.1365-2567.2008.03026.x 19178593 PMC2669810

[B2] Biron C. A. Nguyen K. B. Pien G. C. Cousens L. P. Salazar-Mather T. P. (1999). Natural killer cells in antiviral defense: function and regulation by innate cytokines. Annu. Rev. Immunol. 17, 189–220 10.1146/annurev.immunol.17.1.189 10358757

[B3] Cedar H. Bergman Y. (2009). Linking DNA methylation and histone modification: patterns and paradigms. Nat. Rev. Genet. 10, 295–304 10.1038/nrn2603 19308066

[B4] Chang S. Aune T. M. (2005). Histone hyperacetylated domains across the Ifng gene region in natural killer cells and T cells. Proc. Natl. Acad. Sci. U.S.A. 102, 17095–17100 10.1073/pnas.0503275102 16286661 PMC1283154

[B5] Chang S. Aune T. M. (2007). Dynamic changes in histone-methylation ‘marks’ across the locus encoding interferon-gamma during the differentiation of T helper type 2 cells. Nat. Immunol. 8, 723–731 10.1038/ni1473 17546034

[B6] Chang S. Collins P. L. Aune T. M. (2008). T-bet dependent removal of Sin3A-histone deacetylase complexes at the Ifng locus drives Th1 differentiation. J. Immunol. 181, 8372–8381 19050254 10.4049/jimmunol.181.12.8372 PMC2794428

[B7] Collier S. P. Collins P. L. Williams C. L. Boothby M. R. Aune T. M. (2012). Cutting edge: influence of Tmevpg1, a long intergenic noncoding RNA, on the expression of Ifng by Th1 cells. J. Immunol. 189, 2084–2088 10.4049/jimmunol.1200774 22851706 PMC3424368

[B8] Collins P. L. Chang S. Henderson M. Soutto M. Davis G. M. McLoed A. G. (2010). Distal regions of the human IFNG locus direct cell type-specific expression. J. Immunol. 185, 1492–1501 10.4049/jimmunol.1000124 20574006 PMC2923829

[B9] Collins P. L. Henderson M. A. Aune T. M. (2012a). Diverse functions of distal regulatory elements at the IFNG locus. J. Immunol. 188, 1726–1733 10.4049/jimmunol.1102879 22246629 PMC3273639

[B10] Collins P. L. Henderson M. A. Aune T. M. (2012b). Lineage-specific adjacent IFNG and IL26 genes share a common distal enhancer element. Genes Immun. 13, 481–488 10.1038/gene.2012.22 22622197 PMC4180225

[B11] Dong J. Chang H. D. Ivascu C. Qian Y. Rezai S. Okhrimenko A. (2013). Loss of methylation at the IFNG promoter and CNS-1 is associated with the development of functional IFN-gamma memory in human CD4(+) T lymphocytes. Eur. J. Immunol. 43, 793–804 10.1002/eji.201242858 23255246

[B12] Dumoutier L. Van Roost E. Ameye G. Michaux L. Renauld J. C. (2000). IL-TIF/IL-22: genomic organization and mapping of the human and mouse genes. Genes Immun. 1, 488–494 10.1038/sj.gene.6363716 11197690

[B13] Dunn G. P. Old L. J. Schreiber R. D. (2004). The three Es of cancer immunoediting. Annu. Rev. Immunol. 22, 329–360 10.1146/annurev.immunol.22.012703.104803 15032581

[B14] Eivazova E. R. Aune T. M. (2004). Dynamic alterations in the conformation of the Ifng gene region during T helper cell differentiation. Proc. Natl. Acad. Sci. U.S.A. 101, 251–256 10.1073/pnas.0303919101 14691261 PMC314171

[B15] Filippova G. N. Fagerlie S. Klenova E. M. Myers C. Dehner Y. Goodwin G. (1996). An exceptionally conserved transcriptional repressor, CTCF, employs different combinations of zinc fingers to bind diverged promoter sequences of avian and mammalian c-myc oncogenes. Mol. Cell. Biol. 16, 2802–2813 8649389 10.1128/mcb.16.6.2802 PMC231272

[B16] Gomez J. A. Wapinski O. L. Yang Y. W. Bureau J. F. Gopinath S. Monack D. M. (2013). The NeST long ncRNA controls microbial susceptibility and epigenetic activation of the interferon-gamma locus. Cell 152, 743–754 10.1016/j.cell.2013.01.015 23415224 PMC3577098

[B17] Gupta R. A. Shah N. Wang K. C. Kim J. Horlings H. M. Wong D. J. (2010). Long non-coding RNA HOTAIR reprograms chromatin state to promote cancer metastasis. Nature 464, 1071–1076 10.1038/nature08975 20393566 PMC3049919

[B18] Guttman M. Amit I. Garber M. French C. Lin M. F. Feldser D. (2009). Chromatin signature reveals over a thousand highly conserved large non-coding RNAs in mammals. Nature 458, 223–227 10.1038/nature07672 19182780 PMC2754849

[B19] Hadjur S. Williams L. M. Ryan N. K. Cobb B. S. Sexton T. Fraser P. (2009). Cohesins form chromosomal cis-interactions at the developmentally regulated IFNG locus. Nature 460, 410–413 19458616 10.1038/nature08079 PMC2869028

[B20] Hatton R. D. Harrington L. E. Luther R. J. Wakefield T. Janowski K. M. Oliver J. R. (2006). A distal conserved sequence element controls Ifng gene expression by T cells and NK cells. Immunity 25, 717–729 10.1016/j.immuni.2006.09.007 17070076

[B21] Huarte M. Guttman M. Feldser D. Garber M. Koziol M. J. Kenzelmann-Broz D. (2010). A large intergenic noncoding RNA induced by p53 mediates global gene repression in the p53 response. Cell 142, 409–419 10.1016/j.cell.2010.06.040 20673990 PMC2956184

[B22] Hung T. Wang Y. Lin M. F. Koegel A. K. Kotake Y. Grant G. D. (2011). Extensive and coordinated transcription of noncoding RNAs within cell-cycle promoters. Nat. Genet. 43, 621–629 10.1038/ng.848 21642992 PMC3652667

[B23] Ishii N. Ozaki K. Sato H. Mizuno H. Saito S. Takahashi A. (2006). Identification of a novel non-coding RNA, MIAT, that confers risk of myocardial infarction. J. Hum. Genet. 51, 1087–1099 10.1007/s10038-006-0070-9 17066261

[B24] Janson P. C. Marits P. Thorn M. Ohlsson R. Winqvist O. (2008). CpG methylation of the IFNG gene as a mechanism to induce immunosuppression [correction of immunosuppression] in tumor-infiltrating lymphocytes. J. Immunol. 181, 2878–2886 18684979 10.4049/jimmunol.181.4.2878

[B25] Ji P. Diederichs S. Wang W. Boing S. Metzger R. Schneider P. M. (2003). MALAT-1, a novel noncoding RNA, and thymosin beta4 predict metastasis and survival in early-stage non-small cell lung cancer. Oncogene 22, 8031–8041 10.1038/sj.onc.1206928 12970751

[B26] Kaplan M. H. Schindler U. Smiley S. T. Grusby M. J. (1996). Stat6 is required for mediating responses to IL-4 and for development of Th2 cells. Immunity 4, 313–319 10.1016/S1074-7613(00)80439-2 8624821

[B27] Kerin T. Ramanathan A. Rivas K. Grepo N. Coetzee G. A. Campbell D. B. (2012). A noncoding RNA antisense to moesin at 5p14.1 in autism. Sci. Transl. Med. 4, 128ra140 10.1126/scitranslmed.3003479 22491950

[B28] Klattenhoff C. A. Scheuermann J. C. Surface L. E. Bradley R. K. Fields P. A. Steinhauser M. L. (2013). Braveheart, a long noncoding RNA required for cardiovascular lineage commitment. Cell 152, 570–583 10.1016/j.cell.2013.01.003 23352431 PMC3563769

[B29] Lercher M. J. Urrutia A. O. Hurst L. D. (2002). Clustering of housekeeping genes provides a unified model of gene order in the human genome. Nat. Genet. 31, 180–183 10.1038/ng887 11992122

[B30] Lercher M. J. Urrutia A. O. Pavlicek A. Hurst L. D. (2003). A unification of mosaic structures in the human genome. Hum. Mol. Genet. 12, 2411–2415 10.1093/hmg/ddg251 12915446

[B31] Mosmann T. R. Coffman R. L. (1989). TH1 and TH2 cells: different patterns of lymphokine secretion lead to different functional properties. Annu. Rev. Immunol. 7, 145–173 10.1146/annurev.iy.07.040189.001045 2523712

[B32] Mullen A. C. Hutchins A. S. High F. A. Lee H. W. Sykes K. J. Chodosh L. A. (2002). Hlx is induced by and genetically interacts with T-bet to promote heritable T(H)1 gene induction. Nat. Immunol. 3, 652–658 12055627 10.1038/ni807

[B33] Nagano T. Fraser P. (2011). No-nonsense functions for long noncoding RNAs. Cell 145, 178–181 10.1016/j.cell.2011.03.014 21496640

[B34] Northrop J. K. Thomas R. M. Wells A. D. Shen H. (2006). Epigenetic remodeling of the IL-2 and IFN-gamma loci in memory CD8 T cells is influenced by CD4 T cells. J. Immunol. 177, 1062–1069 16818762 10.4049/jimmunol.177.2.1062

[B35] Okano M. Bell D. W. Haber D. A. Li E. (1999). DNA methyltransferases Dnmt3a and Dnmt3b are essential for de novo methylation and mammalian development. Cell 99, 247–257 10.1016/S0092-8674(00)81656-6 10555141

[B36] Orom U. A. Derrien T. Beringer M. Gumireddy K. Gardini A. Bussotti G. (2010). Long noncoding RNAs with enhancer-like function in human cells. Cell 143, 46–58 10.1016/j.cell.2010.09.001 20887892 PMC4108080

[B37] Phillips J. E. Corces V. G. (2009). CTCF: master weaver of the genome. Cell 137, 1194–1211 10.1016/j.cell.2009.06.001 19563753 PMC3040116

[B38] Rea S. Eisenhaber F. O’Carroll D. Strahl B. D. Sun Z. W. Schmid M. (2000). Regulation of chromatin structure by site-specific histone H3 methyltransferases. Nature 406, 593–599 10.1038/35020506 10949293

[B39] Rinn J. L. Kertesz M. Wang J. K. Squazzo S. L. Xu X. Brugmann S. A. (2007). Functional demarcation of active and silent chromatin domains in human HOX loci by noncoding RNAs. Cell 129, 1311–1323 10.1016/j.cell.2007.05.022 17604720 PMC2084369

[B40] Rubio E. D. Reiss D. J. Welcsh P. L. Disteche C. M. Filippova G. N. Baliga N. S. (2008). CTCF physically links cohesin to chromatin. Proc. Natl. Acad. Sci. U.S.A. 105, 8309–8314 10.1073/pnas.0801273105 18550811 PMC2448833

[B41] Sajan S. A. Hawkins R. D. (2012). Methods for identifying higher-order chromatin structure. Annu. Rev. Genomics Hum. Genet. 13, 59–82 10.1146/annurev-genom-090711-163818 22703176

[B42] Schoenborn J. R. Dorschner M. O. Sekimata M. Santer D. M. Shnyreva M. Fitzpatrick D. R. (2007). Comprehensive epigenetic profiling identifies multiple distal regulatory elements directing transcription of the gene encoding interferon-gamma. Nat. Immunol. 8, 732–742 10.1038/nrg2211 17546033 PMC2144744

[B43] Sekimata M. Perez-Melgosa M. Miller S. A. Weinmann A. S. Sabo P. J. Sandstrom R. (2009). CCCTC-binding factor and the transcription factor T-bet orchestrate T helper 1 cell-specific structure and function at the interferon-gamma locus. Immunity 31, 551–564 10.1016/j.immuni.2009.08.021 19818655 PMC2810421

[B44] Shi M. Lin T. H. Appell K. C. Berg L. J. (2008). Janus-kinase-3-dependent signals induce chromatin remodeling at the Ifng locus during T helper 1 cell differentiation. Immunity 28, 763–773 10.1016/j.immuni.2008.04.016 18549798 PMC2587400

[B45] Shnyreva M. Weaver W. M. Blanchette M. Taylor S. L. Tompa M. Fitzpatrick D. R. (2004). Evolutionarily conserved sequence elements that positively regulate IFN-gamma expression in T cells. Proc. Natl. Acad. Sci. U.S.A. 101, 12622–12627 10.1073/pnas.0400849101 15304658 PMC515107

[B46] Sleutels F. Zwart R. Barlow D. P. (2002). The non-coding Air RNA is required for silencing autosomal imprinted genes. Nature 415, 810–813 10.1038/415810a 11845212

[B47] Soutto M. Zhou W. Aune T. M. (2002). Cutting edge: distal regulatory elements are required to achieve selective expression of IFN-gamma in Th1/Tc1 effector cells. J. Immunol. 169, 6664–6667 12471094 10.4049/jimmunol.169.12.6664

[B48] Spilianakis C. G. Lalioti M. D. Town T. Lee G. R. Flavell R. A. (2005). Interchromosomal associations between alternatively expressed loci. Nature 435, 637–645 10.1038/nature03574 15880101

[B49] Strahl B. D. Allis C. D. (2000). The language of covalent histone modifications. Nature 403, 41–45 10.1038/47412 10638745

[B50] Szabo S. J. Kim S. T. Costa G. L. Zhang X. Fathman C. G. Glimcher L. H. (2000). A novel transcription factor, T-bet, directs Th1 lineage commitment. Cell 100, 655–669 10.1016/S0092-8674(00)80702-3 10761931

[B51] Tato C. M. Martins G. A. High F. A. Dicioccio C. B. Reiner S. L. Hunter C. A. (2004). Cutting edge: innate production of IFN-gamma by NK cells is independent of epigenetic modification of the IFN-gamma promoter. J. Immunol. 173, 1514–1517 15265878 10.4049/jimmunol.173.3.1514

[B52] Thierfelder W. E. Van Deursen J. M. Yamamoto K. Tripp R. A. Sarawar S. R. Carson R. T. (1996). Requirement for Stat4 in interleukin-12-mediated responses of natural killer and T cells. Nature 382, 171–174 10.1038/382171a0 8700208

[B53] Thomas R. M. Gamper C. J. Ladle B. H. Powell J. D. Wells A. D. (2012). De novo DNA methylation is required to restrict T helper lineage plasticity. J. Biol. Chem. 287, 22900–22909 10.1074/jbc.M111.312785 22584578 PMC3391093

[B54] Tian D. Sun S. Lee J. T. (2010). The long noncoding RNA, Jpx, is a molecular switch for X chromosome inactivation. Cell 143, 390–403 10.1016/j.cell.2010.09.049 21029862 PMC2994261

[B55] Tong Y. Aune T. Boothby M. (2005). T-bet antagonizes mSin3a recruitment and transactivates a fully methylated IFN-gamma promoter via a conserved T-box half-site. Proc. Natl. Acad. Sci. U.S.A. 102, 2034–2039 10.1073/pnas.0409510102 15684083 PMC548570

[B56] Tsai M. C. Manor O. Wan Y. Mosammaparast N. Wang J. K. Lan F. (2010). Long noncoding RNA as modular scaffold of histone modification complexes. Science 329, 689–693 10.1126/science.1192002 20616235 PMC2967777

[B57] Turner B. M. (2002). Cellular memory and the histone code. Cell 111, 285–291 10.1016/S0092-8674(02)01080-2 12419240

[B58] Vakoc C. R. Mandat S. A. Olenchock B. A. Blobel G. A. (2005). Histone H3 lysine 9 methylation and HP1gamma are associated with transcription elongation through mammalian chromatin. Mol. Cell 19, 381–391 10.1016/j.molcel.2005.06.011 16061184

[B59] Vigneau S. Rohrlich P. S. Brahic M. Bureau J. F. (2003). Tmevpg1, a candidate gene for the control of Theiler’s virus persistence, could be implicated in the regulation of gamma interferon. J. Virol. 77, 5632–5638 10.1128/JVI.77.10.5632-5638.2003 12719555 PMC154023

[B60] Weaver C. T. Hatton R. D. Mangan P. R. Harrington L. E. (2007). IL-17 family cytokines and the expanding diversity of effector T cell lineages. Annu. Rev. Immunol. 25, 821–852 10.1146/annurev.immunol.25.022106.141557 17201677

[B61] Willingham A. T. Orth A. P. Batalov S. Peters E. C. Wen B. G. Aza-Blanc P. (2005). A strategy for probing the function of noncoding RNAs finds a repressor of NFAT. Science 309, 1570–1573 10.1126/science.1115901 16141075

[B62] Wilson C. B. Makar K. W. Perez-Melgosa M. (2002). Epigenetic regulation of T cell fate and function. J. Infect. Dis. 185(Suppl. 1), S37–S45 10.1086/338001 11865438

[B63] Wutz A. (2011). Gene silencing in X-chromosome inactivation: advances in understanding facultative heterochromatin formation. Nat. Rev. Genet. 12, 542–553 10.1038/nrg3035 21765457

[B64] Yao H. Brick K. Evrard Y. Xiao T. Camerini-Otero R. D. Felsenfeld G. (2010). Mediation of CTCF transcriptional insulation by DEAD-box RNA-binding protein p68 and steroid receptor RNA activator SRA. Genes Dev. 24, 2543–2555 10.1101/gad.1967810 20966046 PMC2975930

[B65] Zheng W. Flavell R. A. (1997). The transcription factor GATA-3 is necessary and sufficient for Th2 cytokine gene expression in CD4 T cells. Cell 89, 587–596 10.1016/S0092-8674(00)80240-8 9160750

[B66] Zhou W. Chang S. Aune T. M. (2004). Long-range histone acetylation of the Ifng gene is an essential feature of T cell differentiation. Proc. Natl. Acad. Sci. U.S.A. 101, 2440–2445 10.1073/pnas.0306002101 14983028 PMC356969

[B67] Zhu J. Yamane H. Paul W. E. (2010). Differentiation of effector CD4 T cell populations (*). Annu. Rev. Immunol. 28, 445–489 10.1146/annurev-immunol-030409-101212 20192806 PMC3502616

